# When two Z-scores meet—analysis of exercise capacity of children and adolescents with Kawasaki disease by a new Z-score model of coronary artery and a new Z-score evaluating peak oxygen consumption: Coronary artery Z-score and peakVO2 Z-score in KD

**DOI:** 10.1186/s13052-023-01535-3

**Published:** 2023-09-29

**Authors:** Sheng-Hui Tuan, Jin-Hui Chung, Guan-Bo Chen, Shu-Fen Sun, I-Hsiu Liou, Chien-Hui Li, Yi-Ju Tsai

**Affiliations:** 1 Institute of Allied Health Sciences, College of Medicine, National Cheng Kung University, Tainan, Taiwan (R.O.C.); 2 Department of Rehabilitation Medicine, Cishan Hospital, Ministry of Health and Welfare, Kaohsiung, Taiwan (R.O.C.); 3 School of Medicine, Kaohsiung Medical University, Kaohsiung, Taiwan (R.O.C.); 4 Department of Physical Medicine and Rehabilitation, Kaohsiung Medical University Chung-Ho Memorial Hospital, Kaohsiung, Taiwan (R.O.C.); 5 Department of Internal Medicine, Kaohsiung Armed Forces General Hospital, Kaohsiung, Taiwan (R.O.C.); 6 Department of Physical Medicine and Rehabilitation, Kaohsiung Veterans General Hospital, Kaohsiung, Taiwan (R.O.C.); 7 School of Medicine, National Yang Ming Chiao Tung University, Taipei, Taiwan (R.O.C.); 8 Department of Physical Therapy, College of Medicine, National Cheng Kung University, Tainan, Taiwan (R.O.C.)

**Keywords:** Cardiopulmonary exercise testing, Coronary artery Z score, Exercise capacity, Kawasaki disease, Peak oxygen consumption

## Abstract

**Background:**

Coronary artery (CA) Z-score system is widely used to define CA aneurysm (CAA). Children and adolescents after acute stage of Kawasaki disease (KD-CA) have a higher risk of developing CAAs if their CA Z-score ≥ 2.5. Z-score system of peak oxygen consumption (Peak VO_2_ Z-score) allows comparisons across ages and sex, regardless of body size and puberty. We aimed to compare the exercise capacity (EC) indicated by peak VO_2_ Z-score during cardiopulmonary exercise testing (CPET) directly between KD-CA with different CA Z-score.

**Methods:**

KD-CA after acute stage who received CPET in the last 5 years were retrospectively recruited. CA Z-score was based on Lambda-Mu-Sigma method. Max-Z was the maximum CA Z-score of different CAs. KD children with Max-Z < 2.5 and ≥ 2.5 were defined as KD-1 and KD-2 groups, respectively. Peak VO_2_ Z-score was calculated using the equation established based on Hong Kong Chinese children and adolescent database.

**Results:**

One hundred two KD-CA were recruited (mean age: 11.71 ± 2.57 years). The mean percent of measured peak VO_2_ to predicted value (peak PD%) was 90.11 ± 13.33. All basic characteristics and baseline pulmonary function indices were comparable between KD-1 (*n* = 87) and KD-2 (*n* = 15). KD-1 had significantly higher peak VO_2_ Z-score (*p* = .025), peak PD% (*p* = .008), peak metabolic equivalent (*p* = .027), and peak rate pressure product (*p* = .036) than KD-2.

**Conclusions:**

KD-CA had slightly reduced EC than healthy peers. KD-CA with Max-Z ≥ 2.5 had significantly lower peak EC than those < 2.5. Max-Z is potentially useful follow-up indicator after acute stage of KD.

## Impact

- Children and adolescents after acute stage of Kawasaki disease (KD-CA) have a higher risk of developing coronary artery (CA) aneurysm if their CA Z-score ≥ 2.5.
- One newly developed Z-score system of peak oxygen consumption (PeakVO2 Z-score) allows comparisons of exercise capacity (EC) across ages and sex for Chinese children and adolescents more accurately and reliably.
- Combining the above-mentioned two Z-score system, KD-CA with CA Z-score ≥ 2.5 had significantly lower peak EC than those < 2.5.
- CA Z-score and PeakVO2 Z-score should be used together as useful follow-up indicators after acute stage of KD.

## Introduction

Kawasaki disease (KD), which was first described in 1967 by Dr. Tomisaku Kawasaki [1], is now the most common form of pediatric vasculitis in children [2]. After Japan and Korea, Taiwan has the third-highest incidence of KD in the world (69 in 1,00,000 children aged < 5 years) [3]. The condition is characterized by systemic inflammation in medium-sized arteries, multiple organs, and tissues during the acute febrile phase, with a predilection for the coronary artery (CA) [4]. An estimated 25% and 5% of untreated and treated children with KD, respectively, may develop CA aneurysms (CAAs) [5]. Although 80% of the CAAs tend to regress within 5 years after the onset of KD, 1% of the CAAs eventually lead to progressive arterial stenosis and thrombosis [6]. These cardiac sequelae may cause myocardial infarction or sudden death in the acute phase, post-acute phase, or even in adulthood [7]. Therefore, both the American Heart Association (AHA) [2] and the Japanese Ministry of Health and welfare [8] recommend routine echocardiographic coronary examination for children with KD to evaluate the presence of CAAs.

Earlier, CAAs were diagnosed based on the CA size as per the Japanese Ministry of Health and Welfare recommendations [9]. These criteria were controversial due to the lack of a correlation with body habitus and the non-differentiation between the right and left CAs. The CA Z-score system, describing how many standard deviations above or below a size or age-specific population mean a given measurement lies [10], can allow more efficient discrimination of CAAs and show better correlation with clinical outcomes [11]. CA Z-score system is derived from a large heterogeneous population of children undergoing echocardiography. Several different CA Z-score regression Eqs. [11], both linear [12] and exponential [13] functions with body surface area (BSA), have been proposed to provide an objective basis for determining CA size abnormalities. In Taiwan, Lin et al. established reference ranges for the CA diameters after evaluating a nationwide cohort of 412 healthy children aged < 6 years [10]. However, there is a lack of available norms for CA for Taiwanese children aged > 6 years. A well-known and acceptable [14, 15] CA Z-score equation for children aged from 0 month to 18.9 years has been established by Kobayashi et al. to estimate the sex-specific Z-score of each internal CA diameter (ZSP version 4) [16]. This equation showed better goodness of fit compared to previously reported regression models. Our team had also used it in our previous study and found that KD children with higher CA Z-score had significantly lower peak exercise capacity (EC) than those with lower CA Z-score [17]. Therefore, we chose ZSP version 4 for our analysis.

In previous studies based on the findings of direct cardiopulmonary exercise testing (CPET), children with a history of KD showed comparable EC [18–20] but lower myocardial flow reserve and higher total coronary resistance compared to their healthy peers [20]. However, adolescents with KD history had significantly lower aerobic metabolism capacity and peak exercise load capacity than their peers [21, 22]. Although, most of the above-mentioned studies used peak oxygen consumption (peak VO_2_), the current gold standard marker of exercise capacity, there are still some limitations that should be addressed when using peak VO_2_. Peak VO_2_ may vary with age, maturity, and sex. It is developmentally divergent and shows a strong correlation with body size and body composition [23]. Conventionally, we scale peak VO_2_ by simply dividing it (mL/min) by body mass (mL/kg/min) to obtain peak metabolic equivalent (peak MET). However, the peak VO_2_ should be scaled for size using the general allometric equation to derive the appropriate size power function (y/xb), thereby providing a more appropriate interpretation of size-related (xb) changes in physiologic function (y) [24]. Recently, a new Z-score equation based on allometric scaled peak VO_2_ values was developed for Southern Chinese children and adolescents (Peak VO2 Z-score). Peak VO_2_ Z-score improves the evaluation of cardiopulmonary fitness, allowing comparisons across ages and sex and will likely provide a better metric for tracking temporal changes in children and adolescents, regardless of body size and age [25]. Therefore, by combining the two Z-scores together, we aimed to compare the difference of cardiopulmonary function indicated by peak VO_2_ Z-score between KD children and adolescents with different CA Z-score in our study.

## Materials and methods

### Subject characteristics

This was a retrospective cohort study conducted at a single center in southern Taiwan. Children and adolescents aged 8–18 years who were referred from the pediatric cardiology outpatient clinic to our rehabilitation department for regular follow-up of KD between January 2017 and February 2022 were recruited. The inclusion criteria were patients who underwent (A) complete transthoracic echocardiographic examination; (B) standard 12-lead electrocardiogram; and (C) symptom-limited treadmill exercise test. The exclusion criteria were: (A) significant structural heart disease; (B) moderate–severe cardiac valvular disease; (C) significant arrhythmia; (D) ventricular hypertrophy; and (E) concurrent pulmonary disease or any other disease that may affect cardiopulmonary function. Basic characteristics including sex, age, body weight, height, and body fat were recorded. The study was conducted following the principles outlined in the Helsinki Declaration and was approved by the Institutional Review Board of Kaohsiung Veterans General Hospital (number: VGHKS17-CT11-11). The study adheres to the STrengthening Reporting of OBservational studies in Epidemiology (STROBE) reporting guidelines.

The sample size estimation was based on the Statistical G*Power software (version 3.1.9.2, for Windows). Considering the study purpose, a two-tailed test with 0.8 effect size, alpha of 0.05, and power of 0.80 [26] with allocation ratio 3:1 (as observed in our previous study [17]) were factored, yielding a sample size of at least 51 and 17 in each group to detect the effect.

### Cardiopulmonary exercise testing and equation of peak oxygen consumption Z-score

All the participants in this study received symptom-limited exercise testing, which was composed of a treadmill, a flow module, a gas analyzer, and an electrocardiographic monitor (Metamax 3B; Cortex Biophysik GmbH Co., Leipzig, Germany). Each participant was familiarized to the procedures and testing equipment through a demonstration by one experienced physical therapist (C.H.C.) before the CPET. The informed consent was obtained after the purpose of the testing was explained to the participants and their families (verbal consent from participants and written consent from family members). We used the Bruce ramp protocol, as suggested by the American College of Sports Medicine (ACSM) and the American Heart Association (AHA) during the CPET [27, 28]. We terminated the test when the participants attained maximal effort according to the ACSM definition, when they demonstrated subjective unbearable symptoms, or when they could no longer continue [28]. The whole test was supervised by a physiatrist with > 20 years of experience in CPET (K.L.L.). The oxygen consumption (VO_2_) and carbon dioxide production (VCO_2_) during the testing was measured using the breath-by-breath method. Blood pressure (BP), heart rate (HR), and respiratory exchange ratio (RER) were also monitored throughout the test. Peak rate pressure product (PRPP), which is an indicator of the myocardial oxygen demand and myocardial workload during exercise, was calculated as the peak systolic BP multiplied by the peak HR [29]. The anaerobic threshold (AT) was measured by using the VE/VO_2_ and VE/VCO_2_ methods [30]. The VO_2_ at the AT (AT VO_2_) and the maximum oxygen uptake measured at peak exercise (peak VO_2_) were determined by the same physiatrist (K.L.L.). The measured VO_2_ was divided by a constant (3.5 mL/kg/min) to derive the MET. The peak VO_2_ Z-score was calculated based on the scaling equation proposed by Yu et al. which is applicable to Chinese children and adolescents by age and sex [24]. Percent of peak VO_2_ to predicted value (peak PD%) was the percentage of measured peak MET to predicted peak MET by reference proposed by Yu et al. after comparing with the normal standards for cardiopulmonary responses to exercise [24]. Both the peak VO2 Z-score and the peak PD% can be obtained using an automated excel file (https://www.ncbi.nlm.nih.gov/pmc/articles/PMC6413916/).

### Pulmonary function test

Pulmonary function test was performed by spirometry at rest. All subjects underwent the pulmonary test after the demonstration and under the guidance of a therapist (C.H.C.). We measured the forced vital capacity (FVC), forced expiratory volume in one second (FEV1), and maximal voluntary ventilation (MVV) and we divided the measured FVC by predicted FVC (FVCP), measured FEV1 by predicted FEV1 (FEV1P), and measured MVV by predicted MVV (MVVP). The predicted value of FVCP, FEV1P, and MVVP were calculated based on the spirometric reference equations for healthy children and adolescence in Taiwan [31].

### Echocardiography and Coronary artery measurements

The echocardiography examination was done in accordance with the AHA scientific statement on KD [2]. All subjects were examined in the supine or left lateral decubitus position by two well-experienced pediatric cardiologists with two-dimensional, M-mode, and Doppler echocardiography (sector probe > 5-MHz frequency). The focus depth was set to the CA measured, and the frame rate was increased to raise the time resolution. CA luminal diameters were measured from inner edge to inner edge in all segments, avoiding points of branching. The right CA (RCA) and left anterior descending CA (LCA) were measured 3–5 mm distal to their origins in the parasternal short-axis view. Routinely examined cardiac structures like valves, left ventricular (LV) diameter, left atrium (LA) and aortic root (AO) diameter, LV diameter at end diastole (LVIDd), and LV diameter at end diastole (LVIDs) were also measured according to the American Society of Echocardiography guidelines and standards for pediatric Echocardiogram [32].

### Equation of coronary artery Z score

The luminal dimensions were assessed by CA Z-score as described by Kobayashi et al. [16]. The CA Z-score was computed by entering sex-specific data on age, body height, body weight, BSA using Haycock formula, and diameter of CA measured by echocardiography in the ZSP version 4 calculator. The largest CA Z-score of proximal LCA or RCA was defined as Max-Z. The subjects were classified depending on the presence or absence of CA aneurysm. Since a mean Z-score > 2.5 usually indicates the presence of CA aneurysm (small aneurysm mean Z-score 2.5 to < 5.0; medium aneurysm 5.0 to < 10; and large aneurysm from ≥ 10 [2]), subjects with Max-Z < 2.5 were defined as KD group 1 and those with Max-Z ≥ 2.5 as KD group 2.

### Statistical analysis

All statistical analysis were conducted using SPSS for Windows version 22.0 (Released 2021. Armonk, NY: IBM Corp). Data were expressed as mean ± standard deviation. Given the relatively smaller number of patients in the KD group 2, Mann–Whitney U test (for continuous variables) or chi square test (for categorical variables) were used to compare the demographic characteristics, pulmonary function, parameters of CPET, and echocardiographic findings between the KD group 1 and the KD group 2. Spearman’s correlation analysis was used to determine the correlation between EC and echocardiographic measurable variables (including CA Z-score). *P* values ≤ 0.05 were considered indicative of statistical significance.

## Results

One hundred twelve patients with KD qualified the inclusion criteria. Of these ten patients were excluded (one each with moderate and severe cardiac valvular disease, two with significant arrhythmia, and six with incomplete pulmonary function test/ PCET data). Therefore, 102 children and adolescents with KD were included in this study. Among them, 87 (85.3%) had CA Z-score < 2.5 (KD group 1) and 15 (14.7%) had CA Z-score ≥ 2.5 (KD group 2).

### Demographic characteristics

Table 1 summarizes the demographic characteristics of the participants. The mean age of all KD patients, patients in KD group 1, and patients in KD group 2 were 11.71 ± 2.57, 11.64 ± 2.56, and 12.15 ± 2.67 years, respectively. There was no significant differences between KD group 1 and KD group 2 with respect to sex, age, weight, height, BMI, body fat, systolic blood pressure (SBP), diastolic blood pressure (DBP), resting HR, or spirometry parameters (including FVC, FVCP, FEV1, FEV1P, MVV, and MVVP). The FVCP, FEV1P, and MVVP of all KD patients (103.26%, 117.18%, and 98.47%, respectively), patients in KD group 1 (103.86%, 119.59%, and 98.37%, respectively), and patients in KD group 2 (98.93%, 99.62%, and 99.36%, respectively) were all more than 80% of the age-predicted value. This indicated that the pulmonary function of children and adolescents with KD was comparable to that of their healthy peers.

**Table 1:** Demographic characteristics of patients with Kawasaki disease

	KD total (*n* = 102)	KD group 1 (*n* = 87)	KD group 2 (*n* = 15)	*P* value ^a^
Gender (M:F)	58:44	51:36	7:8	.404
Age (yr)	11.71 ± 2.57	11.64 ± 2.56	12.15 ± 2.67	.503
Height (cm)	148.76 ± 16.30	148.64 ± 15.87	149.59 ± 19.73	.844
Weight (kg)	46.15 ± 16.62	46.00 ± 15.59	47.18 ± 23.26	.813
BMI (kg/m^2^)	20.14 ± 4.19	20.17 ± 4.04	19.97 ± 5.30	.876
Resting SBP (mmHg)	112.78 ± 15.00	112.97 ± 14.91	111.54 ± 16.20	.750
Resting DBP (mmHg)	67.40 ± 9.45	67.04 ± 9.42	69.85 ± 9.66	.321
Resting HR (bpm)	84.66 ± 12.23	84.13 ± 11.99	88.23 ± 13.78	.262
FVC (L)	2.58 ± .97	2.54 ± .94	2.84 ± 1.21	.376
FVCP (%)	103.26 ± 24.76	103.86 ± 25.93	98.93 ± 13.51	.558
FEV1 (L)	2.25 ± .80	2.22 ± .78	2.46 ± .97	.379
FEV1P (%)	117.18 ± 13.71	119.59 ± 13.69	99.62 ± 17.43	.635
FEV1/FVC (%)	87.73 ± 10.51	87.75 ± 10.88	87.60 ± 7.66	.968
MVV (L)	61.99 ± 26.12	60.41 ± 22.86	74.39 ± 44.39	.378
MVVP (%)	98.47 ± 44.10	98.37 ± 42.30	99.36 ± 61.28	.953

Data are the mean ± standard deviation

*KD* Kawasaki disease, *KD group 1* Max-Z of coronary artery less than 2.5, *KD group 2* Max-Z of coronary artery equals to or more than 2.5, *BMI* body mass index, *SBP* systolic blood pressure, *DBP* diastolic blood pressure, *HR* heart rate, *FVC* functional vital capacity, *FVCP* percentage of predicted forced vital capacity, *FEV1* force expiratory volume at 1 min, *FEV1P* percentage of predicted forced expiratory volume at 1 min, *MVV* maximal voluntary ventilation, *MVVP* percentage of predicted maximal voluntary ventilation

^a^ Refers to the *p* value of a Mann–Whitney U test (continuous variables) or chi square test (categorical variables) between KD group 1 and KD group 2

### Comparisons of performance of treadmill exercise testing

Table 2 shows the performance of exercise test of all recruited KD participants, the KD group 1, and the KD group 2, respectively. The mean peak RER of all KD patients, patients in KD group 1, and patients in KD group 2 were 1.16 ± 0.09, 1.16 ± 0.09, and 1.11 ± 0.09, respectively. This indicated that almost all KD patients could reach maximal effort in the CPET. KD children and adolescents had lower EC as compared to their peers since the peak VO_2_ Z-score and peak PD% of all KD participants was − 0.60 ± 0.95 and 90.11 ± 13.33%, respectively.

**Table 2:** Performance of exercise test in all patients with Kawasaki disease, KD group 1, and KD group2

	KD total (*n* = 102)	KD group 1 (*n* = 87)	KD group 2 (*n* = 15)	*P* value ^a^
Peak VO_2_ Z-score	- .60 ± .95	- .52 ± .92	- .15 ± .99	.025*
Peak PD% (%)	90.11 ± 13.33	91.44 ± 12.47	81.04 ± 15.95	.008*
AT MET	6.84 ± 1.22	6.91 ± 1.23	6.38 ± 1.09	.146
AT HR (bpm)	141.59 ± 13.61	141.33 ± 13.10	143.38 ± 17.23	.613
peak MET	10.07 ± 1.62	10.21 ± 1.55	9.15 ± 1.89	.027*
peak HR (bpm)	179.21 ± 10.43	179.71 ± 9.87	175.77 ± 13.65	.205
peak VE (L)	45.79 ± 14.83	46.57 ± 14.67	40.51 ± 15.45	.170
peak RER	1.16 ± .09	1.16 ± .09	1.11 ± .09	.066
peak SBP (mmHg)	160.86 ± 30.55	163.11 ± 31.12	145.46 ± 21.42	.051
peak DBP (mmHg)	79.06 ± 19.40	77.67 ± 18.65	88.54 ± 22.49	.059
PRPP	28,899.12 ± 6051.85	29,378.21 ± 6108.51	25,619.15 ± 4611.54	.036*
HRR at 1 min	29.66 ± 11.86	30.18 ± 12.03	25.44 ± 9.94	.261

Data are the mean ± standard deviation

*Peak VO*_*2*_* Z-score* Z-score of peak oxygen consumption based on reference value, *Peak PD%* measured peak oxygen consumption divided by predicted peak oxygen consumption, *KD* Kawasaki disease, *KD group 1* Max-Z of coronary artery less than 2.5, *KD group 2* Max-Z of coronary artery equals to or more than 2.5, *MET* metabolic equivalent, *AT MET* MET at the point of anaerobic threshold, *peak MET* largest MET during whole exercise testing, *HR* heart rate, *peak PD* percentage of predicted peak MET, *VE* minute ventilation, *peak RER* largest respiratory exchange ratio during whole exercise testing, *SBP* systolic blood pressure, *DBP* diastolic blood pressure, *PRPP* peak rate pressure product, *HRR at one minute* heart rate reserve at one minute after termination of the test

*p < .05

^a^ Refers to the *p* value of a Mann–Whitney U test between KD group 1 and KD group 2

**Fig. 1:**
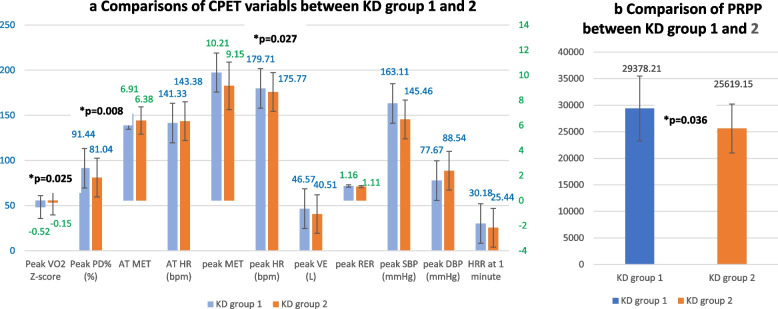
Comparisons of the performance of exercise test between Kawasaki group 1 and 2. Legend: Among all the routined measured variables during cardiopulmonary exercise testing, KD group 1 had significantly higher **a** peak VO2 Z-score, peak PD%, peak MET, and **b** PRPP than the KD group 2. Peak VO_2_ Z-score, Z-score of peak oxygen consumption based on reference value; Peak PD%, measured peak oxygen consumption divided by predicted peak oxygen consumption; KD, Kawasaki disease; KD group 1: Max-Z of coronary artery less than 2.5; KD group 2: Max-Z of coronary artery equals to or more than 2.5; MET, metabolic equivalent; AT MET, MET at the point of anaerobic threshold; peak MET, largest MET during whole exercise testing; HR, heart rate; peak PD, percentage of predicted peak MET; VE, minute ventilation; peak RER, largest respiratory exchange ratio during whole exercise testing; SBP, systolic blood pressure; DBP, diastolic blood pressure; PRPP, peak rate pressure product; HRR at one minute, heart rate reserve at one minute after termination of the test

As for the comparisons between the KD group 1 and the KD group 2, KD group 1 had significantly higher peak VO_2_ Z-score (− 0.52 ± 0.92 vs. − 1.15 ± 0.99, *P* = 0.025), peak PD% (91.44 ± 12.47% vs. 81.04 ± 15.95, *P* = 0.008), peak MET (10.21 ± 1.55 vs. 9.15 ± 1.89, *P* = 0.027), and PRPP (29,378.21 ± 6108.51 vs. 25,619.15 ± 4611.54, *P* = 0.036) than the KD group 2. All the other routine parameters measured during the CPET, including MET at the point of AT (AT MET), HR at the point of AT, peak DBP, and heart rate reserve at one minute after termination of the test (HRR), showed no significant difference between the two groups (Fig. 1).

### Echocardiographic findings

Table 3 shows the echocardiographic findings of all KD patients, the KD group 1, and the KD group 2. The proximal RCA Z-score, proximal LCA Z-score, and Max-Z of all recruited participants were 0.03 ± 1.14, 1.40 ± 1.05, and 1.45 ± 1.00, respectively. In the KD group 1, the proximal RCA Z-score, proximal LCA Z-score, and Max-Z were 0.15 ± 0.99, 1.13 ± 0.77, and 1.17 ± 0.70, respectively. In the KD group 2, the proximal RCA Z-score, proximal LCA Z-score, and Max-Z were 2.04 ± 0.54, 3.26 ± 0.78, and 3.36 ± 0.67, respectively. All the routinely examined echocardiographic parameters, including LVIDd, LVIDs, LV shortening fraction, diameter of LA and AO, showed no significant difference between the KD group 1 and the KD group 2.

**Table 3:** Echocardiographic findings of children and adolescence with Kawasaki disease

	KD total (*n* = 102)	KD group 1 (*n* = 87)	KD group 2 (*n* = 15)	*P* value ^a^
LVIDd (cm)	3.99 ± .66	4.01 ± .61	3.83 ± .93	.130
LVIDs (cm)	2.36 ± .45	2.39 ± .44	2.20 ± .52	.115
LV shortening FR (%)	40.34 ± 11.94	40.06 ± 12.61	42.23 ± 5.53	.634
LA (cm)	2.04 ± .32	1.99 ± .36	2.09 ± .42	.335
AO (cm)	1.78 ± .33	1.75 ± .40	1.89 ± .63	.257
RCA diameter (cm)	.27 ± .06	.28 ± .04	.32 ± .08	.016*
LCA diameter (cm)	.30 ± .06	.27 ± .05	.40 ± .07	< .001*
RCA Z score by ZSP	.03 ± 1.14	.15 ± .99	2.04 ± .54	< .001*
LCA Z score by ZSP	1.40 ± 1.05	1.13 ± .77	3.26 ± .78	< .001*
Max-Z by ZSP	1.45 ± 1.00	1.17 ± .70	3.36 ± 0.67	< .001*

Data are the mean ± standard deviation

*KD* Kawasaki disease, *KD group 1* Max-Z of coronary artery less than 2, *KD group 2* Max-Z of coronary artery equals to or more than 2, *LVIDd* diastolic left ventricular (LV) internal diameter, *LVIDs* systolic left ventricular internal diameter, *LV shortening FR* LV shortening fraction = (LVIDd-LVIDs)/ LVIDd, *LA* diameter of left atrium, *AO* diameter of aortic root, *RCA* right coronary artery measured 3 to 5 mm distal to its origins in the parasternal short-axis view, *LCA* left anterior descending coronary artery measured 3 to 5 mm distal to its origins in the parasternal short-axis view, *RCA/LCA Z score by ZSP* coronary Z score of RCA/LCA calculated by ZSP version 4 calculator, *Max-Z by ZSP* maximum Z-score of the LCA or RCA by ZSP version 4 calculator

*p < 0.05

^a^ Refers to the *p* value of a Mann–Whitney U test between KD group 1 and KD group 2

## Discussion

To the best of our knowledge, this is the first study to compare the peak VO_2_ Z-score between KD children and adolescents with different CA Z-scores. We found that after the acute stage of KD, although most KD children and adolescents were able to exert sufficient effort to reach peak performance during CPET, there was still mildly decreased peak PD% as compared to normal reference. We also observed that KD children and adolescents with MAX-Z < 2.5 had higher peak EC, including peak VO_2_ Z-score, peak PD%, peak MET, and PRPP compared to those with MAX-Z ≥ 2.5.

Few studies have investigated the exercise performance and aerobic capacity in patients with KD. Allen et al. used CPET with leg ergometer to evaluate the performance of KD patients. They found no difference in total work rate, mean power, maximal HR, and maximal VO_2_ between KD children and control subjects [33]. In the study by Rhodes et al. (1996), participants performed the CPET via leg ergometer and they found that KD patients had similar maximal VO_2_, peak workload, and AT compared to the control participants [34]. Wang et al. (2008) observed that KD children had similar maximal HR but lower maximal VO_2_ and maximal SBP compared to healthy peers during the CPET with treadmill [34]. Our previous study also found no significant difference in the aerobic metabolism and peak exercise load capacities of KD children and control group [20]. However, the healthy controls in all of the studies mentioned above were collected from previously reported normal participants from the database of each institution, matched for sex, age, and BMI, or BSA. Therefore, the results may have been affected by potential selection and population bias. In the present study, the mean peak PD% of all the KD children and adolescents was 90.11 ± 13.33%. Given that peak PD% was the percentage of measured peak MET to predicted peak MET by an age- and sex-specific reference established based on a large cohort of Southern Chinese children and adolescents, we believe that the results may be more accurate than the above-mentioned studies. KD children and adolescents had relatively lower peak MET than their healthy peers. The results of a recent study by Yang et al. who recruited age- and sex-matched healthy volunteers through poster advertising were consistent with our results; they reported that adolescents with KD history had significantly lower peak VO_2_/kg (approximately 7.93%) than controls [21].

In our previous study, we also found that the PRPP of KD patients was significantly lower than that of controls and the Max-Z of CA showed a significant inverse correlation with PRPP [20]. These findings indicated that KD patients may still have compromised coronary perfusion during exercise after the acute stage and it is crucial to examine the impact of pathological change in CA with EC among KD patients. However, there are few studies about the EC of KD patients with different CA Z-score or CA aneurysm. Allen et al. found no differences in maximal voluntary work rate and maximal VO_2_ between KD patients with and without aneurysms [33]. Paridon et al. divided the KD children and adolescents into three groups (one group with no objective evidence of CAA, one group with resolved CAA, and the other one with persistent CAA) and found that the maximal VO_2_ was normal after acute KD regardless of the status of CA [35]. Both studies were conducted before 1995, when the idea and application of CA Z-score was not mature and commonly used. The presence of CAA was directly recognized by cardiologist via echocardiography or angiography. Our previous study used ZSP version 4 to calculate CA Z-score and found that children with KD who had higher Max-Z had significantly lower peak MET and PRPP than those with a lower Max-Z. Consistent with our previous study, in the present study, children and adolescents with Max-Z ≥ 2.5 had significantly higher peak VO_2_ Z-score and peak PD% than those with Max-Z < 2.5. Since the allometric scaling peak VO_2_ Z-score equations were developed for different sex and age groups, which were effective in removing the influence of body mass, height, and age on peak VO_2_, these findings indicate that the effects of KD on the cardiovascular system persists for years after the acute stage, which might influence the cardiopulmonary function of patients with KD. Moreover, given that PRPP is a good indicator of coronary flow reserve, it is plausible that the weaker performance during CPET may have resulted from the compromised coronary perfusion during peak exercise in KD participants with Max-Z ≥ 2.5. We assumed that the difference in peak EC between the KD group 1 and 2 might be attributed to CA-related factors such as, CA endothelial dysfunction, compliance of CA lumen, and different CA resistance to inflammatory status [18].

Indeed, the long term effects of KD on the circulatory system may persist even without the presence of CAA. Iemura et al. performed ultrasound cardiography and Tsuchihashiet al. used selective coronary angiography (CAG) to examine the structural changes in CA after the acute stage. Both these studies demonstrated the presence of an abnormal vascular structure even though small CAAs in the acute phase of KD reverted to a normal appearance in the convalescent phase at the previous site of an acute CAA [36, 37]. Some reports have described patients with angiographically normal CA after acute KD who later developed cardiovascular disorders in their early adulthood [38, 39]. Therefore, regular follow-up of KD patients with echocardiography and CPET after the acute stage is crucial.

The Z-score describes how many standard deviations above or below a size or age-specific population mean a given measurement lies. In the context of KD, both western (2017 AHA [2]) and eastern (2020 Japanese Circulation Society Joint Working Group [8]) guidelines recently have endorsed the use of CA Z-score system to define coronary abnormalities and classify CAAs. CA Z-score systems were shown to improve risk classifications of CAAs and predict the clinical prognosis [11]. The application of peak VO_2_ Z-score for better evaluation of cardiopulmonary fitness is a new concept [24, 25]. It is important to eliminate the effect of body size on CPET parameters to obtain body size–independent reference values in children and adolescents whose aerobic capacity are strongly influenced by body size and pubertal stage [40]. Adequate peak VO_2_ Z-score equation is independent of body size and pubertal stage. Use of peak VO_2_ Z-score allows us to compare across ages and sex and might provide a better metric for tracking changes over time [25]. By combining these two concepts of Z-score in this current study, we found that KD children and adolescents with MAX-Z < 2.5 had higher peak VO_2_ Z-score than those with MAX-Z ≥ 2.5. This result was in accordance with our previous study showing that KD children with higher Max-Z had significantly lower peak MET [17]. Moreover, it provides a more definitive evidence of the influence of KD on the EC since the peak VO_2_ Z-score is independent of sex, age, body size, puberty, and BMI.

Last but not the least, the author wanted to emphasize that KD populations should still engage in exercise normally even though there might be compromised CA flow during the peak exercise effort. The RER is defined as the ratio of VCO_2_ and VO_2_ consumption measured via respiratory gas analysis. Peak RER ≥ 1.10 is considered the minimal requirement to perform sufficient effort during CPET [28]. The peak RER in all KD participants was 1.16 ± 0.09. Even the participants in the KD group 2 had peak RER of 1.11 ± 0.09. This means that most KD children in our study, irrespective of KD-1 or KD-2 group, could reach peak exercise testing value. Moreover, a physical activity requiring more than 6 METs is considered vigorous according to the definition of ACSM, and the average peak MET in the KD group 1 and 2 of our study were 10.21 ± 1.55 and 9.15 ± 1.89, respectively. There findings suggest that all the KD participants in our study may engage in normal vigorous daily activities.

Some limitations of this study should be considered. First, this was a retrospective study. Although we tried our best to perform the CPET soon (within days) after complete transthoracic echocardiographic examination, there was still some variability in the timing of follow-up. Second, the number of KD patients who had Max-Z ≥ 2.5 (*n* = 15) was lower than the minimum required sample size (*n* = 17). Although this distribution was in line with the previous study [5, 7], the results should be viewed in light of this limitation. Third, the subjects were recruited from a single medical center in southern Taiwan. A larger cross-national study is required for further evaluation. Last, the ZSP version 4 is an equation based on the data from Japan. Given the current lack of a well-accepted equation for CA Z-score based on Taiwanese children aged > 6 years, the ZSP version 4 might be the most appropriate and available one. However, the Max-Z by ZSP version 4 calculator may not fully reflect the CA condition of Chinese KD children and adolescents. This situation also applies to peak VO_2_ Z-score. Although the peak VO_2_ Z-score equation used in this study was derived from data of Southern Chinese children and adolescents, there might be still differences between the Taiwanese and Cantonese.

## Conclusion

In this study, KD children and adolescents showed slightly reduced peak EC than their healthy peers even though they could reach maximal exercise effort during CPET. In addition, KD children and adolescents with Max-Z < 2.5 had higher peak EC, including peak VO_2_ Z-score, peak PD%, peak MET, and PRPP, than those with MAX-Z ≥ 2.5. Incorporating both the CA Z-score and peak VO_2_ Z-score, we could offer clinicians a more precise tool to evaluate cardiopulmonary fitness in children and adolescents, irrespective of age and body size. This metric can lead to enhanced clinical decisions for KD patients, by providing a comprehensive view of cardiopulmonary health. Moreover, our results of peak EC might be due to CA difference since the pulmonary function indices were comparable in the two groups. It is important to promote cardiovascular health of all KD patients after the acute stage owing to the potential long term pathological effects on CA. Monitoring the cardiovascular risk of KD children with Max-Z ≥ 2.5 is imperative.

## Data Availability

Individual participant data that underlie the results reported in this article, after deidentification might be shared. Proposals should be directed to Gabbrile@vghks.gov.tw. for application.

## Abbreviations

KD: Kawasaki disease
CA: Coronary artery
CAA: Coronary artery aneurysm
AHA: American Heart Association
EC: Exercise capacity
CPET: Cardiopulmonary exercise testing
peak VO_2_: Peak oxygen consumption
peak MET: Peak metabolic equivalent
ACSM: American College of Sports Medicine
VCO2: Carbon dioxide production
BP: Blood pressure
HR: Heart rate
RER: Respiratory exchange ratio
peak RPP: Peak rate-pressure product
AT: Anaerobic threshold
AT VO_2_: VO_2_ at the AT
FVC: Forced vital capacity
FEV1: Forced expiratory volume in one second
MVV: Maximal voluntary ventilation
FVCP: Percentage of predicted forced vital capacity
FEV1P: Percentage of predicted forced expiratory volume at 1 min
MVVP: Percentage of predicted maximal voluntary ventilation
RCA: Right coronary artery measured 3 to 5 mm distal to its origins in the parasternal short-axis view
LCA: Left anterior descending coronary artery measured 3 to 5 mm distal to its origins in the parasternal short-axis view
LV: Left ventricular
LA: Left atrium
AO: Aortic root
LVIDd: LV diameter at end diastole
LVIDs: LV diameter at end diastole
Max-Z: Largest coronary artery Z-score of proximal left anterior descending artery or right coronary artery
